# Assessing community readiness for overweight and obesity prevention in pre-adolescent girls: a case study

**DOI:** 10.1186/1471-2458-13-1205

**Published:** 2013-12-20

**Authors:** Joanna May Kesten, Noel Cameron, Paula Louise Griffiths

**Affiliations:** 1 Centre for Global Health and Human Development, Loughborough University, LE11 3TU, Loughborough, UK

**Keywords:** Community readiness, Obesity prevention, Pre-adolescent girls

## Abstract

**Background:**

Childhood overweight and obesity is a global public health concern. For girls in particular, being overweight or obese during pre-adolescence (aged 7–11 years) has intergenerational implications for both the mother and her future offspring. In the United Kingdom (UK) there is increasing interest in community targeted interventions but less is known about how to tailor these approaches to the needs of the community. This study applied the Community Readiness Model (CRM), for the first time in the UK, to demonstrate its applicability in designing tailored interventions.

**Methods:**

Community readiness assessment was conducted using semi-structured key informant interviews. The community’s key informants were identified through focus groups with pre-adolescent girls. The interviews addressed the community’s efforts; community knowledge of the efforts; leadership; community climate; community knowledge of the issue and resources available to support the issue. Interviews were conducted until the point of theoretical saturation and questions were asked separately regarding physical activity (PA) and healthy eating and drinking (HED) behaviours. The interviews were transcribed verbatim and were firstly analysed thematically and then scored using the assessment guidelines produced by the CRM authors.

**Results:**

Readiness in this community was higher for PA than for HED behaviours. The lowest scores related to the community’s ’resources’ and the ’community knowledge of the issue’; affirming these two issues as the most appropriate initial targets for intervention. In terms of resources, there is also a need for resources to support the development of HED efforts beyond the school. Investment in greater physical education training for primary school teachers was also identified as an intervention priority. To address the community’s knowledge of the issue, raising the awareness of the prevalence of pre-adolescent girls’ health behaviours is a priority at the local community level. Inconsistent school approaches contributed to tensions between schools and parents regarding school food policies.

**Conclusions:**

This study has identified the readiness level within a UK community to address the behaviours related to overweight and obesity prevention in pre-adolescent girls. The focus of an intervention in this community should initially be resources and raising awareness of the issue within the community.

## Background

Childhood overweight and obesity represent significant public health concerns [1,2]. Despite evidence supporting a stabilisation in the prevalence of children with excess adiposity both in England [2] and in other European countries, Australia, Japan and the USA [3,4], the levels are still of concern [3-5]. There is evidence for a causal association between childhood overweight and obesity and the risk factors associated with the development of chronic diseases in later life [1,6]. In particular, there are inter-generational implications for the future of women as mothers when they are overweight and obese during pre-adolescence (aged 7–11 years) [7-9]. Children who gain excessive amounts of weight during childhood, particularly girls, have an increased risk of overweight in their offspring, independent of parental adult BMI levels [9,10]. Therefore, research investigating the formation of lifestyle choices which contribute towards energy balance behaviours in pre-adolescent girls is warranted.

A systematic review of interventions that aim to prevent overweight and obesity in pre-adolescent girls suggests that interventions should include a broader range of social settings than the school and family supporting the potential effectiveness of approaches which focus on community settings (incorporating families, schools and the environment) [11]. Support for the importance of the environment’s influence on health comes from ecological models of health behaviour which advocate acknowledging that individuals, their health and their environment are interdependent [12]. The United Kingdom (UK) Coalition government advocates that community targeted approaches “which work best for local people” are required (p6) [13]. However less is known about how these targeted approaches can be achieved in an affordable and sustainable way. Given the potential importance of community and environmental factors that contribute to behaviour change, the Community Readiness Model (CRM) may be an effective means of developing tailored community-level behaviour change interventions. The CRM proposes the integration of a “community’s culture, resources and level of readiness” [14] to effectively address community issues. “Readiness” refers to the preparedness of a group to “take action on an issue” (p3) and can predict the likelihood that change will be achieved and supported by a community [14]. Prevention programmes utilising local people’s knowledge and resources, to solve local issues, effectively encourage ownership of the programme by local people, which is an important element of programme success [15] and sustainability. In support of the CRM’s utility, the new NICE guidelines for working with local communities in efforts to tackle obesity advocate engaging the community to identify local priorities and the most appropriate actions to address these priorities [16]. Throughout this engagement process local networks, including key members who can support efforts, should be identified and involved in the development of initiatives [16].

The CRM development has been described in more detail elsewhere [17]. Briefly the model has developed from two research traditions: Psychological Readiness and Community Development (Innovative Decision Making Model and Social Action Process) [17,18]. To incorporate these theories into an assessment of community readiness, key informant interviews are employed because it is believed that within every community there will be people with extensive knowledge about the issue under study and the ability to suggest ways of tackling the problem [17]. Anchored rating (short) statements describing a series of “critical incidents” needed to perform a certain task and the stages of readiness were produced through expert consultation and a modified Delphi procedure (p667) [17]. During this process it was decided that the anchored rating statements needed to be divided across five dimensions: community efforts and community knowledge of the efforts; leadership (support for initiatives from influential community members); community climate (attitude of the community towards the issue); community knowledge of the issue; and resources. These dimensions form the interview guide sections. To the authors’ knowledge, the CRM is the only model designed to assess readiness at the community level.

The CRM has been applied to childhood overweight and obesity prevention previously in America and Australia to do the following: calculate a pre-intervention readiness score and implement strategies to prevent overweight and obesity in childhood [19]; identify a community with a readiness score high enough to implement a community-based obesity prevention intervention in 6–9 year olds through a competitive process; [20] and assess readiness pre and post a community-based obesity prevention intervention conducted with 12–18 year olds [21]. The latter found that intervention schools increased their readiness, whilst control schools did not, and those intervention schools with the greatest increases had the greatest decreases in the prevalence of overweight and obesity [21]. Lastly, the Ready for Recess study [22] has published findings suggesting that community readiness may be related to the effectiveness of a physical activity intervention in 8–12 year olds which the authors conclude means community readiness should be raised to a level where the community is likely to accept the need for change prior to intervention.

The aim of the current research is to be the first in the UK to assess the stage of community readiness to prevent overweight and obesity in pre-adolescent girls by focusing primarily on the behaviours related to excess weight gain (Physical activity [PA] and Healthy Eating and Drinking [HED]). The results from this research will inform the design of a tailored community intervention.

## Methods

This research was conducted in the Charnwood Borough in Leicestershire, within the East Midlands Region of the UK. The Charnwood Borough was selected as the community for this study because: it has not previously engaged in a similar community-based programme; it is geographically contained; and it is an appropriate size (approximately 166,100 inhabitants [23]) for conducting community research as it is neither so large that there is no sense of community nor so small that a successful intervention would have little wider public health implications. Demographically it is comparable to the English population of 7–11 year olds. For example, in 2011/12, the prevalence of overweight and obesity in 10–11 year old children was similar to the rest of England (32% and 33.9% respectively) [24].

Community readiness assessment was conducted using semi-structured key informant interviews following guidelines adapted from the CRM authors [14]. The interview guide has six sections corresponding to the model’s dimensions: community efforts; community knowledge of efforts; leadership; community climate; community knowledge of the issue; and resources available to support the issue [14,15,17,25]. PA and HED were considered to be two distinct behaviours with different factors influencing whether individuals perform them, so the interview questions were repeated once for PA and once for HED. The participants were asked to limit their responses to the discussion of 7 to 11 year old girls rather than children in general.

To identify the key informants who influence PA and HED behaviours in pre-adolescent girls, thirteen focus groups were conducted with 56 girls aged between 6–11 years in 8 schools [26] representative of schools in the Charnwood Borough in terms of school size and area level deprivation measured using the Index of Multiple Deprivation (IMD). The IMD is a relative measure of deprivation comprised of 7 domains: income; employment; health deprivation and disability; education; skills and training; crime; and living environment [27]. Before the focus groups took place, parents and children provided written informed consent and assent respectively.

From the focus groups, the following key informants were identified: celebrities; doctors; dentists; dinner staff; grandparents; Government; girl-guide leaders; head teachers; neighbours; parents; the peer group; sports coaches; siblings; school cooks; shop-keepers; and teachers. The following key informants were considered ineligible for interview: celebrities; dentists; doctors; grandparents; neighbours; siblings; and the peer group. Celebrities do not meet the CRM’s definition of a key informant as they are unlikely to know about the community situation [14]: instead questions were added to the interview guide regarding the influence of the media which incorporated celebrities. Doctors and dentists were not interviewed because it was more relevant to keep the focus on community members rather than community health professionals. In relation to the siblings and the peer group, it was decided that it would be too challenging for children to answer the interview questions and too difficult to recruit older siblings. Recruiting parents was difficult so it was decided that trying to recruit grandparents, through the school or parents, would not be feasible and would add additional burden to the participating parents. Instead, interview questions were included which addressed the influence of grandparents, the peer group and siblings, on which other key informant family members could comment. Neighbours, in the focus groups were often parents of the peer group; therefore interviewing parents was deemed sufficient.

All schools that had participated in the focus group study were invited to participate in this study. Five, out of the original eight, schools agreed to participate in the key informant interviews and were given information letters to hand out to: parents; teachers of 7 to 11 year olds; teaching assistants; school cooks; and school dinner staff. During the initial interviews with parents the participants were mainly from medium to least deprived backgrounds (as indicated by their occupations and comments made in the interview). In order to recruit representatives from most deprived groups a second round of school recruitment then focused on recruiting schools in most deprived areas. This approach recruited a further three parents. The school key informants were classified by IMD level of the school area. However, for parents, the National Statistics-Socio-economic Classification (NC-SEC) [28], produced through self-reported assessment of occupation, was also included. The reason for using the NC-SEC is that both the area and the individual socio-economic classification are likely to be important in influencing parenting practices. For the 16 key informants recruited from outside the school setting, participants were recruited through information letters, emails and telephone calls. On two occasions, sampling followed a snowball technique whereby after or during the interview another possible key informant was mentioned. When this occurred, the researcher asked for the name and contact details of the new key informant and followed this up.

Prior to the key informant interviews, conducted between February and November 2011, informed consent was sought from those who agreed to participate. The ethical advisory committee at Loughborough University approved this research project (R10- P10) on the 24/03/2010.

The CRM’s authors [14] suggest that interviewing 4–6 key informants achieves an understanding of a community issue because each key informant is asked to talk about the wider community context. However, typically within qualitative research, investigators aim to reach a point of ‘theoretical saturation’ whereby no new concepts are expected to be gained by conducting more interviews [29]. In this sense the research is more driven by the data than by any preconceived views of the community situation that the researcher may have. In addition, more than six key informants were identified by the focus group participants. For these reasons this research sought to achieve ‘theoretical saturation’ and recruited more than the suggested number of key informants. ‘Theoretical saturation’ at the community level was assessed iteratively using thematic analysis of each transcript in sequence to detect whether any additional themes were present by comparison with the previous transcript. Thus saturation was reached when little new information or themes were contributed by the last transcript [29].

The interviews were transcribed verbatim by the same researcher (JMK) who conducted the interviews and subsequently analysed the transcripts. A thematic analysis [30] was completed to familiarise the researcher with the transcripts and to develop an in-depth understanding of the key informants’ attitudes and experiences using QSR N-Vivo 8. An in-depth qualitative analysis also aided the interpretation of the readiness scores and enabled the identification of priority areas for intervention in this community. The thematic analysis [30] involved systematically (line by line) grouping the transcripts into initial codes representing basic features of the data. The content of codes was read and compared across other codes to iteratively refine, collapse and group codes into potential themes. Themes were reviewed by all authors.

The transcripts were then scored using the assessment guidelines produced by the CRM authors [14]. For each dimension in the interview guide there are 9 anchored rating statements [14]. The transcripts were systematically read to compare each anchored statement to the transcripts. If there was evidence pointing to the interview transcript meeting the first statement the researcher moved to the next statement, this process continued until an anchored rating statement was reached which was not reflected in the transcripts. The previous statement’s number was then recorded and the next dimension’s statements were analysed. Once all of the dimensions for every interview transcript had an anchored rating score, a mean was calculated by totalling each dimension across all the interviews and dividing by the number of interviews [14]. The mean ratings for each dimension were then added together and divided by the number of dimensions to produce an overall community readiness score. Limited resources meant that the transcripts were not scored independently by two researchers as recommended by in the CRM guidelines [14], however in situations where the scorer (JMK) was unsure about how to code a statement, discussions were held with a second researcher (PLG) until consensus was reached.

## Results

Thirty-three key informant interviews, lasting between 19 minutes and 1 hour 35 minutes, were conducted. Key informant participant characteristics are presented in Table 1. Ten parents (2 male) were recruited from six schools. One interview was conducted as a paired interview with two parents at the participants’ request. Although no parents from schools within the least deprived category were recruited, the parents who participated are considered representative of a diverse range of socio-economic groups as determined by the NC-SEC derived from self-reported occupations [28]. Only one mother was of South Asian Indian origin and Hindu religion and all other parents were of White British origin. The teachers and teaching assistants who agreed to participate worked within schools in the least, medium and most deprived areas. The Healthy Schools Advisor was responsible for supporting and monitoring schools once they have achieved the National Healthy School Status. This UK government initiative [31] focuses on four key areas: Personal Social, Health and Economic Education; Healthy Eating; Physical Activity and Emotional Health and Wellbeing. Although no school cooks agreed to participate in this study, an interview with the School Food Advisor who oversees the School Meal Support service represented them.

**Table 1:** Key informant participant characteristics

**Key informant**	**Subcategory or description of characteristics (number of schools)**	**Total**	Subcategory (NS-SEC)*	**Total**	**Overall total**
Parents	Least deprived school	0	Managerial and professional occupations	2	
	Medium deprived school (2)	2	Intermediate occupations	3	
			Small employers and own account workers	1	
			Lower supervisory and technical occupations	0	
			Semi-routine and routine occupations	1	
			Unemployed	3	
	Most deprived school (4)	8			10
Teachers and teaching assistants**	Least deprived school (1)	1			
	Medium deprived school (3)	3			
	Most deprived school (1)	2			6
Dinner staff	Most deprived school	1			1
Government initiative leaders	Healthy Schools Advisor	1			
	School Food Advisor	1			2
Shop-keepers	Urban area	1			
	Rural area	1			2
Girl guide leaders	Based on a diverse range of geographical settings across the community	6			6
Sports coaches	Sports Development Officers	2			
	Football Development Officer	1			
	Gymnastics Coach	1			
	Community Sports Coach/Play Ranger	1			6
	School Sports Coach	1			
TOTAL N:					33

*National Statistics Socio-economic Classification [28] derived from self-coded method of self-reported occupation into one of five classes. **Two of the teaching assistants were also dinner staff.

### Community readiness model scores

The mean PA readiness score was 6.08 which corresponds to the ‘Initiation Stage’ (Table 2). This stage is reached when ‘enough information is available to justify efforts and activities are underway’ [14]. The mean HED readiness score was 5.74, corresponding to the ‘Preparation Stage’. At this stage active community leaders have begun planning efforts by deciding what should be done and by whom and the community offers modest support. The readiness scores for community efforts were the highest of all the dimensions for both HED and PA. The resources score is the lowest for the HED behaviours and the second lowest readiness score for PA suggesting initiatives are limited by the available resources. The lowest readiness score concerning PA is in the dimension ‘community knowledge of the issue’ , suggesting that low PA levels in pre-adolescent girls are not viewed as an issue in this community. The following section focuses on potential intervention strategies within each dimension which could increase the community readiness scores and prevent overweight and obesity amongst pre-adolescent girls.

**Table 2:** Community readiness scores

**Dimensions**	**Community efforts**	**Knowledge of community efforts**	**Leadership**	**Community climate**	**Knowledge of the issue**	**Resources**
Physical activity						
Total score	235	188	226	178	163	177
Mean score (standard deviation)	7.34 (0.97)	5.88 (1.44)	7.06 (1.19)	5.56 (1.76)	5.13 (1.21)	5.53 (0.97)
Overall readiness stage	6.08 (INITIATION STAGE)					
Healthy eating and drinking						
Total score	229	187	214	181	168	143
Mean score (standard deviation)	6.94 (1.43)	5.67 (1.73)	6.48 (2.14)	5.48 (2.06)	5.09 (1.13)	4.77 (0.42)
Overall readiness stage	5.74 (PREPARATION STAGE)					

### Community efforts

The community efforts identified by the key informants are presented in Table 3. Generally, the initiatives had been in place for several years and the majority were conducted within schools.

**Table 3:** Community efforts to support healthy eating and drinking (HED) and physical activity (PA)

**Type of initiative**	**Name**	**Description**
Community based	Family Lifestyle Club (FLIC)	Eight-week targeted intervention for families with an overweight or obese child or those that would benefit from additional support identified through GP practices [32].
	Girl Guides	Guides included working towards achieving health related badges such as the Healthy Heart and cookery badge.
	Change 4 Life	*“I think you know social marketing campaigns have helped you know, Change 4 Life etc.”* Healthy Schools Advisor
	School Food Support service	*“We put [school] menu’s together. We work very closely with the county dietician. (…) We also talk to school councils. So we actually talk to the children themselves (…) I also talk to head teachers about improving lunch time experiences for children.”* School Food Advisor
	Holiday PA programmes	Held in two local leisure centres.
	Adopt a school	*“We offer them [a primary school] the leisure centre, for free of charge (…) because they haven’t got a sports hall, (…), so they’ll do activities (…), we’ve done football, cricket, basketball, badminton, tennis, we’re possibly looking at gymnastics. (…) I get a coach to coach the first term (…) I’ll go through what I’m going to do on my 6 weeks lesson plan [with the teacher], (…) and then the following six weeks, I clear off and I let the teacher then, carry it on (…) so the idea is not only are we providing professional coaching for the children (…) but we’re up-skilling the teachers.”* Sports Development Officer
	Wild Card	*“If you live in the Charnwood Borough and you’re aged between 5 and 16 you can apply for a Wild Card. It gives you discounts in certain places it’s not just sport but (…) the cinema (…).”* Parent, Most Deprived School
	Dance classes such as cheerleading	
	Girls’ football	
	Real Planning	*“[someone had] graffiti’d (…) swear words all over the walls so parents are refusing to take their kids there [park], (…) so that will be one of the things that the (…) [real planning leaders] are going to (…) to get cleaned off (…).”*School Sports Coach
Family Based	Cook and Eat session	*“(…) the lady from the Adult Learning Service she demonstrated cooking and preparing (…) a healthy meal which they [families] then had to do at the same time and that was brilliant (…). We have had parents who would never engage with school before who were like ‘oh yeah I’ll come and do that.’”* Teaching Assistant, Medium Deprived school
School based	Breakfast club	
	Life buses	The Life Bus came to schools and covered a range of issues relating to health including diet [33].
	Food detectives	*“We did food detectives (…) some of the older children, were (…) coming into the hall when the younger ones were eating and looking what was in their lunch box. Well sort of delving through it or whatever (…) and checking that they’d got a healthy lunch box and then they were giving out stickers (…) eyes on what’s a healthy lunch*
		*R: how do you think that worked?*
		*It worked well (…) they like that, because they like stickers”* Teaching Assistant, Medium Deprived School
	Allotments/gardening club	
	School fruit scheme	
	School food policy	*“We went down a route of saying ‘ok at break if they bring an unhealthy snack, they will be asked to have it at lunch time’ (…) ‘it’s saying to them that it’s ok to have it sometimes during the day and you know a moderate (…) amount of it but not all the time.”* Deputy Head Teacher, Medium Deprived School
	Afterschool clubs	
	Physical Education	
	Multi-sports	*“It’s more sort of fun based stuff, I suppose, rather than sports specific”.*Football Development Officer
	Cycling proficiency lessons	
	Trim Trail	Wooden climbing frame
	Walk to school schemes	
	Sports day	
	Recreational times	
	Little Leaders	*“The year 6’s they lead it, so I’ve trained them to be leaders and then they lead it so (…) and it just gives them really good skills for sort of later on in life and like prepares them for sort of the little leadership courses they can do when they get to secondary school”. *School Sports Coach
	National healthy school status	*“The Healthy Schools programme is like the umbrella really (…) that brings everything [health policies] together”*
	School Councils	Some schools had formed councils composed of *“two [child] representatives from every year”*, who met to *“discuss what they would like to see promoted under healthy eating”*. School Food Advisor

The readiness score for community efforts could be increased for HED by firstly implementing initiatives in settings which promote PA. This potential intervention target is illustrated by the contradiction of having vending machines stocked with unhealthy food and drink in a community leisure facility which promotes PA.

“There’s the big vending machines with chocolate in for them, when you’ve gone swimming, which seems a little silly.”

#### Parent, Most Deprived School

However, the Sports Development Officer who worked in this leisure centre was reluctant to develop HED efforts because he felt that “*we can give advice but then giving advice can lead to law suits.”*

Secondly, inter-school consistency in approaches to HED policies across this community could be improved. Whilst some schools prohibited unhealthy snacks except for one ‘treat day’ a week, others allowed a small unhealthy snack every lunch time. One school was considering implementing efforts and was in consultation with parents and another school was unsure whether it should tell parents what to do.

“There is quite a concern about whether we [school] have any right to start telling people [parents] what they should and shouldn’t be putting in their children’s lunch boxes.”

#### Deputy Head Teacher, Least Deprived School

To ensure compliance with school HED policies, re-iteration of their importance and reminders for parents are necessary, particularly after school holidays.

“We promote [in schools] the healthy eating and we do particularly for the younger children, we do give stickers out

R: ok so that’s every lunch time?

It’s up in the air again at the minute because it’s after half term again and parents [think], ‘oh shove this chocolate in, you’ll be fine, have these crisps”

#### Teaching Assistant, Medium Deprived School

Whilst some parents disliked constant reminders from schools, others appreciated the compulsory nature of school policies because it alleviated pressure on parents to provide children with unhealthy items. Poor compliance with school HED efforts was explained by the perception that it is easier for parents to give children what they like, which was perceived to be unhealthy food.

“It’s easier for parents to push rubbish into their [lunch] box than think about it.”

#### Teaching Assistant, Medium Deprived School

Improving the PA community efforts could begin by targeting those children who tend not to attend them and *“engender[ing] more participation”* (Deputy Headteacher, Least Deprived School). The sports coaches commented that the same children repeatedly attend community efforts. These children were perceived to be generally more active than those who do not attend and the Community Sports Coach suggested efforts should be tailored to the needs of the least active children. For example, working parents may have limited time to transport children to different activities, reflecting support for activities which occur immediately after school.

“[There’s a] number of children who actually don’t get the chance to participate just because they haven’t got the transport or parents are working.”

#### Deputy Head Teacher, Least Deprived School

To engender participation, it is important to consider individual preferences for different physical activities, for example the Football Development Officer described offering football which he suspected might not be the preferred activity in which girls would want to take part and others described girls particularly enjoying dance.

“It’s about really knowing whether [we’re] offering the right things because obviously we’re offering football to them but again is that one of the major things they [girls] want to play?”

The dinner staff key informant described the problem of girls being inactive at break time and added that if she said to the girls, *“right you can go in [to] school, put some music on and you can dance, they would do it.”* Therefore, enabling girls of this age to make their own choices was viewed as important for the development of preferences for active pursuits.

### Community knowledge of the efforts

The majority of key informants reported high community awareness of the efforts. However, low parental engagement with schools was identified as a barrier to community knowledge of the efforts. This would suggest a need for improved communication between schools and parents.

Three ‘word-of-mouth’ methods for communicating information between schools and parents were identified: ‘parent networks’; ‘child to child’; and ‘school to parent via the child’. One deputy head teacher commented that children may be able to educate parents via the latter communication method.

“Children will take the messages that you’ve given about healthy eating or smoking home to parents and say ‘look, this is what we’ve learnt’ and nag at the parent and that is part of schools’ role and I think sometimes that’s the best way to approach it without getting parents’ backs up.”

#### Deputy Head Teacher, Medium Deprived School

However, some parents discussed disapproval of this method of communication.

“Friends of mine had comments on the children’s lunch box when maybe just once they’ve put in a piece of birthday cake from a family member and it’s been frowned upon and they felt a little bit judged that maybe you know that they always do things like that.

R: … how is that addressed, do teachers speak to the parents?

P: I think that’s one of the bug-bears (…) that people have said that the children have had the odd comment and the parents have been cross that they haven’t been spoken to about it.”

#### Parent, Most Deprived School

In addition, written communication, sending letters to parents, detailing the available activities, was not always viewed as effective.

“Obviously through school, they get letters, so it’s every child gets a letter, it’s whether it makes it home. I’ve not always read all of them. I think parents are guilty of that.”

#### Parent, Most Deprived School

Given these perceptions, it would seem that there is a need for more direct communication and collaboration between schools and parents.

### Leadership

The appointed leaders or influential people in this community were predominantly supportive of PA and HED behaviours. However, leadership support for PA in particular, could be improved. Firstly, one deputy head teacher commented that his community lacked key leaders for PA promotion.

“People of my age are the people who seem to be carrying sport on rather than younger people who are coming into the [teaching] profession and my worry is that in 10 or 12 years’ time I don’t see a natural successor to take on the role that I take on across other schools. And if it wasn’t for me constantly sending out emails and things, I don’t see anyone else in that group who’d do it.”

#### Deputy Head Teacher, Most Deprived School

This same teacher provided an explanation for this lack of leadership stating that the increasing demands on teachers’ time means that they have less time available to run after-school clubs; and when they do give their time to these activities they resent the low child participation.

“There is that feeling, sometimes, of you put on a club and only 5 kids want to come along, is it worth your giving up 45 minutes of your time? Which means those 45 minutes have got to be found at another time to catch up with what you’ve missed.”

Secondly, primary school teachers were not always very enthusiastic or confident about teaching P.E, although this was not a universal opinion.

“There’s no P.E. teachers in particular [who are] specialists in primary schools which I think is a massive problem so the quality varies massively, some teachers are really enthusiastic and some just go through the motions.”

#### Community Sports Coach/Play ranger

The School Sports Coach’s role within schools was to *“provide either PPA [teacher’s planning time] cover or actually, do the P.E. lesson.”* The School Sports Coach believed coaches and teachers took different approaches to teaching P.E. lessons.

“Teachers just don’t want to put that bit extra into it, they’ve got like a check list, ‘we’ve got to get them doing, this, this and this in order to meet national curriculum level 4.’ Whereas I think coaches, which is definitely a positive of the sports coaches, because I’m always telling them ‘yep that was better because you got a better swing on that, that’s why the ball went further’. So we can pick at those technical points and give them that to feed on”

#### School Sports Coach

Although the School Sports Coach offered teachers an opportunity to gain additional expertise, those with whom she worked often would not assist her and would leave the room.

“I don’t think they really enjoy doing P.E. I get that feeling, they’re just like ‘yeah you take them.’”

#### School Sports Coach

### Community climate

The community was perceived to view unhealthy dietary consumption as a ‘reward’ or ‘treat’ and acceptable as long as it is a small component of children’s diet or the consequence of time restrictions.

“It’s a treat to eat unhealthily but if you do that three or four times a week it’s just it comes into the general life.”

#### Sports Development Officer

The move away from traditional nuclear families towards a situation where children potentially spend less time with individual family members was viewed as contributing to the attitude that HED is a treat.

“I think it’s become more acceptable that if they see a parent once a week, ‘I’ll treat you to McDonalds.’”

#### Sports Development Officer

The consumption of unhealthy ‘fast food’ was viewed as convenient and the presence of this behaviour within the community was evidenced by one teacher’s observation that children were unable to eat with a knife and fork.

“It’s quicker to give them something that comes out the microwave or take them to a fast food place. One of the really surprising things over the last few years is the number of children who actually don’t know how to use a knife and fork. I think this gives a picture of what, might be going on at home - the fact they don’t actually sit down and eat properly very often, a lot of it is finger food.”

#### Deputy Head Teacher, Least Deprived School

The School Food Advisor described situations where parents had been angry about school HED policies. For example, school provided meals included desserts which were perceived to be unhealthy whilst in some cases children bringing packed lunches were banned from consuming any unhealthy snacks. The School Food Advisor defended the school meal’s tolerance of desserts because they were providing a nutritionally balanced diet.

“*But desserts aren’t necessarily unhealthy no, so everything that’s on the school menu, it’s balanced over the fortnight’s menu so there aren’t too many sugars in there. There aren’t too many fats.”*

#### School Food Advisor

In addition the Healthy Schools Advisor believed that parents did not object to children eating healthily, but that they objected to the child’s school dictating to them.

*“There are some parents who don’t want to be told you know what they should be giving their children to eat in their packed lunch. So I get quite a few phone calls from irate parents.*”

#### Healthy Schools Advisor

By improving the relationship between parents and schools and developing HED policies in consultation with parents the community climate readiness score could be increased.

“*I don’t know whether they ask very much for the views of parents. The lunch boxes - that was just sort of announced that this is what we’re going to do so it’s not always as collaborative as maybe it could be.”*

#### Parent, Most Deprived School

Whilst most key informants reported that the community would not tolerate inactivity unless a child was unwell, low physical fitness in girls was felt to indicate a degree of tolerance by a variety of key informants such as parents, sports coaches and a deputy head teacher.

“I’d say there should not be [situations where physical inactivity is tolerated] but I think there clearly are. The majority of children are, I would say grossly unfit, can’t take part in sustained exercise for very long at all.”

#### Deputy Head teacher, Least Deprived School

A preference for convenience was also perceived to mean inactivity was tolerated.

“They’ve [primary school] set up this ‘park and stride’. You park at the church and walk up the last hundred yards. Not many people use it because they’re too lazy because that would be walking that little bit further.”

#### Parent, Medium Deprived School

Tolerance of inactivity within schools was justified for two reasons: the weather (meaning teachers do not want to supervise children outside) and the national curriculum (resulting in pressures on academic performance and a tendency to value curriculum subjects included in national assessments more than PA).

“It’s a bit cold today we won’t go out, or we’re a bit busy, or we’ll carry on with this for a bit longer and so things start to get a little bit shrunk.”

#### Deputy Head teacher, Least Deprived School

Some girls in the community, particularly as they reach 10 to 11 years of age were perceived to develop a negative self-perception of their abilities in PA and become reluctant to participate.

“They don’t perceive themselves as a ‘sporty person’ so they don’t really want to take part in sport because they’re worried what other people might think.”

#### Football Development Officer

In addition, some girls were felt to view PA as not being *“particularly cool”* (Girl Guide Leader).

“Year 6 [10–11 year olds] girls in particular will just sit around, because it’s like they’re too cool for that school now so they are in a transition stage. All they do is sit on a bench and chat.”

#### Teaching Assistant, Medium Deprived School

### Community knowledge about the issues

There was mixed awareness amongst community members about the causes and consequences of unhealthy eating and drinking and low PA.

There appeared to be two categories of parental knowledge. The first described performing healthy behaviours with their family and as a result perceived messages to promote child health as not directed at them.

“I don’t necessarily take an awful lot of note of what the government are saying because I think the government aren’t necessarily talking to me.”

#### Parent, Most Deprived School

The second category reported that parental knowledge of HED was poor.

“If you look at our school there are a lot of overweight girls and I don’t think always there is the knowledge [of] and the association between healthy eating and the size that these children are. I think there’s sometimes the perception that the children are just like that because that’s how they’re built.”

#### Parent, Most Deprived School

In relation to this comment about the levels of overweight and obesity in the community, one teacher provided anecdotal evidence that this problem was getting worse by talking about the size of P.E. kit he had to purchase.

“For the first time ever I’ve had to order adult sized bibs for children to do P.E in because they can’t get in them anymore and they can’t wear them and that’s boys and girls.”

#### Deputy Head teacher, Least Deprived School

This study found that the understanding of PA guidelines could be improved amongst ‘experts’ and parents. Guidelines were perceived to be difficult to understand firstly because they were perceived to be constantly changing.

“The government keep changing, (…) so they’ll say that a child of that age needs to be active, they need to [do] moderate activity, half-an-hour a day and then they’ll change it to one hour every week.”

#### Sports Development Officer

The interpretation of guidelines was problematic because individuals were unsure how to assess different intensities of activities without the use of PA monitors.

“What constitutes moderate and what constitutes severe activity? (…) Say if you slightly raise your heart rate? What do I tell a kid, ‘Have you slightly raised your heart rate?”

#### Sports Development Officer

Others had no awareness of the recommended levels of PA and instead adopted a ‘common sense approach’ to monitoring children’s PA (e.g. tiring children out).

Indeed, the knowledge and awareness of the prevalence of PA and HED behaviours in pre-adolescent girls in the community was considered to be limited, suggesting that even if guidelines were understood the community would not be able to accurately assess whether girls are meeting them. The local authority key informants described having statistics relating to these behaviours but did not disseminate or publicise these to the public beyond placing information on websites.

### Resources related to the issue

Resources were available in this community to support HED and PA efforts but were subject to political priorities. Funding for programmes was reported to be predominantly *“cyclical”*. In addition, a change in government resulted in funding priorities shifting towards schools taking responsibility for their own Healthy School status.

“The next stage which is Healthy Schools Enhancement is about focusing on a school priority that’s particularly pertinent for their school and actually do some work in a little bit more depth to actually achieve some meaningful outcomes around health and wellbeing priorities. Quite a few schools for example have chosen things like obesity so, you know, working towards achieving some meaningful outcomes around PA and also healthy eating in order that they can actually do their bit to you know trying to prevent obesity.”

#### Healthy Schools Advisor

Government funding cuts resulted in a feeling of resentment from the Healthy Schools Advisor.

“We’ve gone from a situation where we have the Every Child Matters agenda which is basically about children’s wellbeing, to a very kind of like traditional, idea about, the main things that are important are passing exams and discipline in the classroom. So I’m just hoping that schools will be able to sort of resist you know letting health and wellbeing fall off the agenda.”

Some key informants, reported evaluating the community efforts they provided, although publicising these findings was limited. For example, the school holiday PA efforts, provided by the local authority were evaluated and the results of this evaluation were used to make improvements.

“We had an evaluation session after the summer about how we [coaches] thought the programme had gone with the kids and what we thought worked well. So they are getting everyone’s opinions, because we sent out a poll to parents as well.”

#### Gymnastics Coach

However, the results from this evaluation were then displayed in an area not often seen by the community.

One Sports Development Officer reported evaluating the geographical areas from which children who attended activities had come from.

“We send all the postcodes where the kids have been from to Charnwood Borough Council. We then try and map where the people have come from. So you look at the areas and go ‘Right there’s no one coming from this bit. Have we not marketed to them, if so why didn’t it work?’”

In some instances relatively little evaluation had been applied, although one key informant considered evaluation a good idea for the future. Where no evaluation was conducted, low participation in community efforts resulted in a sense of helplessness because the reasons for non-attendance could not be elicited. Time restraints, particularly for voluntary organisations, were given as a reason for not performing in-depth evaluation of initiatives.

“I don’t do it [child and teacher evaluations] anymore because it takes too long, by the time I’ve processed everything. It’s a totally voluntary organisation that people do and, at the end of the day, I want to encourage, rather than discourage.”

#### Deputy head teacher, least deprived school

## Discussion

This study, assessing the community readiness to address overweight and obesity prevention in pre-adolescent girls, is the first in the UK to apply the CRM. The overall readiness stage achieved for HED (5.74) was the ‘Preparation Stage’ and the PA score (6.53) reached the ‘Initiation Stage’. At the ‘Preparation Stage’ the CRM suggests the community has active leaders who are planning efforts to which the community offers modest support whilst at the ‘Initiation Stage’ information is available to justify the efforts in place. The qualitative analysis aids the interpretation of the higher community readiness stage for PA than for HED behaviours, suggesting that this could be due to the limited HED initiatives operating beyond the school setting and the disagreements between schools and parents regarding school HED policies. The general consensus suggests that HED efforts are more contentious than PA efforts. The two highest scoring dimensions for both HED and PA are: ‘community efforts’ and ‘leadership’ , which suggests this community has implemented initiatives to address energy balance behaviours and has members willing to lead and support these initiatives. The CRM authors state that readiness should be at approximately the same level for every dimension before overall efforts can be successful [14]. Efforts therefore should be focused initially on the lowest scoring dimensions. Given the readiness scores for each dimension, addressing the ‘resources’ dimension, especially for HED efforts, and the ‘community knowledge about the issue’ dimension would be the most appropriate initial targets for intervention because these represent the lowest readiness scores.

### Addressing ‘resources’

The HED score for ‘resources’ (4.77) suggests that the ‘community has individuals, organisations, and space available that could be used as resources’ and for PA (5.53) ‘some members of the community are looking into the available resources’ [14].

The lack of sustainable, (i.e. long-term) funding highlights the need for community members to be encouraged to take ownership of initiatives so that when funding is removed they can build on the commitment and find ways to continue, for example through fund-raising. This recommendation is in line with the principles of Community-based Participatory Research [34].

Resources are also required within this community to enhance the teaching skills of non-specialist primary school teachers, who may lack enthusiasm [35,36]. A recent report from Ofsted suggested that whilst P.E. teaching was considered good in the majority of schools, subject-specific knowledge could be improved and schools need to ensure that children are improving their physical fitness and are being sufficiently active in these lessons [37]. In this community, it had been anticipated that the employment of sports coaches would improve the provision of P.E in schools. However, the impact of this initiative was limited because the teachers with whom the School Sports Coach worked, did not assist in the P.E. lessons. A combined teaching effort from the School Sports Coach and teachers should be encouraged to help improve subject specific skills in P.E.

There is also a need for resources to support the development of HED efforts beyond the school, for example in leisure centres. Survey results support the findings of the current study regarding the unhealthy food and drink provided in Leisure Centres [38]. One of the Sports Development Officers commented that he did not have adequate expertise to give nutritional advice. This highlights the need for HED key informants, such as the School Food Advisor, to work beyond schools within local community settings and for those already working in community settings to receive training in areas with which they are less familiar in relation to HED. A pilot initiative to improve the healthy options available within Leisure Centres concluded that the most appropriate strategy is ‘choice-based’ whereby healthy options are offered alongside more unhealthy options and educational strategies are used to encourage people to make the healthiest choice [39].

### Addressing ‘community knowledge of the issue’

In the dimension ‘community knowledge of the issue’ , both the HED (5.09) and PA (5.13) scores met the anchored rating statement: ‘community members know that the signs and symptoms of this issue occur locally and general information is available’ [14]. When this score is combined with the qualitative analysis of the transcripts it is recommended that efforts be made to raise the community’s awareness of the prevalence of pre-adolescent girls’ behaviours and weight status at the local community level because a community level statistic about the proportion of children meeting recommended levels of PA and HED practices may resonate more with individuals than a national statistic or guideline. However, providing education will not effectively encourage behaviour change without the realisation that change is necessary. For instance, a key issue with parents, as noted in this study, is their ability to recognise what is ‘overweight’ and the behaviours related to increased risk of overweight (e.g. insufficient activity levels) in themselves and their children [40-42]. If parents cannot recognise overweight and its associated behaviours in their children then public health messages aimed at promoting a healthy weight status may be ignored and they may not seek professional support [41,43].

The community’s knowledge of the issue within the Charnwood Borough might also be increased if the evaluations of local initiatives were publicised more [14,17]. For example, if community members know that those attending initiatives found them to be enjoyable, others may also be encouraged to participate.

The methods used by schools to communicate with parents in this community were mainly indirect (written and oral messages delivered by the children, websites and notice boards). By using these indirect methods of communication, schools appeared to view children as ‘vectors’ who are capable of carrying information regarding health home to their parents which was expected to subsequently change parental behaviours. The expectation that children can influence family behaviour, has been critiqued elsewhere because it does not acknowledge the agency children have over their own behaviours and assumes children need others to change their behaviours for them [44]. Some parents in this study disapproved of indirect communication via children because they would prefer teachers to speak directly to them. When information relates specifically to the behaviour of parents, efforts should be made by schools to communicate directly with parents, particularly regarding the provision of food as this seemed more contentious than PA related communication. The logistics of disseminating information to parents directly (in person) was complicated by parents’ occupations, making it difficult to schedule a time which suited all parents. This has been found in another qualitative study [45] and is rarely addressed or incorporated into intervention design.

This study found that inconsistent school approaches contributed to conflicts between schools and parents regarding school meals and packed-lunch policies. Whereas school food standards are legislated [46], schools can design their own policies for packed lunches. This research suggests that the School Food Support service needs to consider becoming involved in ‘whole school’ HED policies to ensure consistency.

Parents also appeared to dislike being told what to do by schools. Similar attitudes regarding HED being a family concern have been found elsewhere [19,47]. Therefore, it is essential that efforts be made to work with parents in school initiatives [48]. A qualitative study addressing the most appropriate way to involve parents in obesity prevention efforts found that most parents believed the level of parental involvement with schools is ‘about right’ [49]. This has implications for interventions which are seeking to increase parents’ involvement because asking parents to do more may not be well-received. To involve parents and children with health education, the feasibility of homework tasks has been assessed [49]. Kipping et al. found that, although most children and parents completed and enjoyed the homework tasks with health education messages, there was limited acceptance that behaviours needed to change beyond completing the tasks because children were perceived to be healthy already [49].

In summary, to increase this community’s readiness to prevent overweight and obesity in pre-adolescent girls, it is recommended that efforts be made to raise awareness of pre-adolescent girls’ health behaviours and the prevalence of overweight and obesity. To achieve sustainable initiatives, communication and collaboration between different settings within and beyond the school should aim to incite community engagement and ownership over initiatives.

### Strengths

The qualitative analysis of the transcripts is a strength of this research because it provides detailed evidence supporting the CRM scoring and identifies the specific needs of the community. Previous publications applying the CRM have not presented a detailed qualitative analysis [19-21,50,51]. The use of focus groups to identify the key informants and the aim of reaching theoretical saturation, adds credibility to this study. The number of interviews conducted was greater than the recommended 4–6, which inevitably utilises more resources. However, it is argued that a fuller, more conceptually representative, understanding of the community has been captured, therefore the authors would recommend that others using the model also consider conducting interviews to theoretical saturation. The authors are only aware of one previous study using the CRM which identified key informants from the target participants’ perspective [52]. By neglecting to identify key informants from the target population’s perspective all key informant types may not be interviewed [20]. In addition, whilst others applying the model have focused on overweight and obesity as the issue under study [19-22], this study is unique in its investigation of the readiness for PA and HED initiatives separately, allowing recommendations to be formed for each behaviour.

### Limitations

Qualitative research relies on contextual factors therefore it must be acknowledged that what is presented in this study is subject to the researchers’ interpretation of what a sample of key informants, from one area in the UK, were willing to talk about at one particular point in time. The lack of an ethnically diverse sample is limiting in this study because the population of Charnwood is diverse with some areas comprised of up to 29.5% Asian or Asian British groups [53]. Although it is not within the scope of this article to critique the CRM in detail, in brief, the scoring of qualitative data to produce a readiness stage has the potential to lose the richness of the data. However in this study the emphasis of the qualitative data has addressed this issue. Due to limited resources, the coding of the transcripts was not conducted by two independent researchers, however coding of which the scorer (JMK) was uncertain was discussed with a second researcher (PLG) until consensus was achieved.

## Conclusion

The Community Readiness Model assessment suggests that this community is in the ‘Initiation’ and ‘Preparation’ stage for physical activity and healthy eating and drinking respectively. When combined with the qualitative component of this model, this assessment has provided the formative basis for designing a tailored intervention to meet the needs of the community. This study has demonstrated the utility of the Community Readiness Model for designing tailored community interventions.

## Abbreviations

CRM: Community readiness model; HED: Healthy eating and drinking; PA: Physical activity; P: Participant; R: Researcher.

## Competing interests

The authors declare that they have no competing interests.

## Authors’ contributions

JMK carried out the interviews, transcribed and analysed the transcripts and led the drafting of the manuscript. NC conceived the research, supervised the data collection and contributed to the drafting of the manuscript. PLG supervised the data collection, contributed to the analysis of the data and contributed to the drafting of the manuscript. All authors read and approved the final manuscript.

## Pre-publication history

The pre-publication history for this paper can be accessed here:

http://www.biomedcentral.com/1471-2458/13/1205/prepub
